# Intra- and inter-day effects of novel robot-assisted hand movement training in individuals with post-stroke hemiparesis: a single-arm pilot study

**DOI:** 10.20407/fmj.2025-025

**Published:** 2026-02-28

**Authors:** Kazuki Ushizawa, Shintaro Uehara, Akiko Yuasa, Taiki Yoshida, Kyoichi Tomita, Takayuki Ohtomo, Shigeo Tanabe, Yohei Otaka

**Affiliations:** 1 Department of Rehabilitation Medicine, School of Medicine, Fujita Health University, Toyoake, Aichi, Japan; 2 Graduate School of Health Sciences, Fujita Health University, Toyoake, Aichi, Japan; 3 Faculty of Rehabilitation, School of Health Sciences, Fujita Health University, Toyoake, Aichi, Japan; 4 Japan Society for the Promotion of Science, Chiyoda, Tokyo, Japan

**Keywords:** Stroke, Rehabilitation, Robotics, Hand, Feasibility studies

## Abstract

**Objectives:**

To investigate the feasibility of robot-assisted hand movement training using a novel end-effector robot in individuals after stroke.

**Methods:**

Eleven individuals with subacute stroke with hand motor impairment underwent robot-assisted repetitive finger flexion/extension for 20 min daily and repeated this training on 7 non-consecutive days. The robot was designed to allow the flexion and extension of the metacarpophalangeal and proximal interphalangeal joints of the index to the little fingers, and to provide assistive torque if the movement did not reach the target angle within a limited time. We assessed the co-contraction index (CCI) of the flexor digitorum superficialis and extensor digitorum muscles and assessed the active range of motion (AROM) of the index finger before and after training each day (intra-day effect). We performed clinical assessments of motor function and spasticity and evaluated the CCI and AROM before and immediately after the 7-day training (inter-day effect).

**Results:**

Ten participants completed the 7-day training. For the intra-day effect, the CCI was significantly decreased immediately after training, particularly during active finger flexion, and the AROM tended to improve from the middle of the training days. For the inter-day effect, there were no significant changes in the Stroke Impairment Assessment Set for Finger Function, modified Ashworth scale, CCI, or AROM after the 7-day training.

**Conclusions:**

Repetitive finger movement training with the assistance of the novel robot improves muscle activation patterns, reducing co-activation between the agonist and antagonist muscles immediately after training.

## Introduction

Stroke is a leading cause of disability in adults, with approximately 12.2 million new cases reported worldwide annually.^1^ One of the primary impairments after stroke is motor paralysis, and approximately 80% of individuals experience upper-extremity hemiparesis post-stroke.^2^ In particular, motor paralysis of the hand is associated with complex symptoms such as spasticity and muscle co-activation. These complex hand motor impairments cause decreased dexterity and coordination of finger motor control, significantly affecting the performance of daily activities such as eating, grooming, and dressing.^3^

Previous studies have shown that high-dose training is crucial for improving motor impairments in stroke rehabilitation.^4,5^ However, in clinical settings, the amount of training performed in post-stroke rehabilitation is lower than that performed in animal studies investigating recovery processes in the nervous system with the repetition of rehabilitation training after stroke.^6–8^ This is possibly because post-stroke rehabilitation for humans cannot focus only on functional training aimed at improving motor impairments, but also includes training for daily activities to generalize the current motor function to the capacity of daily motor performance.^9,10^ In addition, it is challenging to implement high-dose upper-limb training, particularly for individuals with stroke with severe finger motor impairments. These patients usually have difficulty performing voluntary flexion-extension finger movements, which hinders their participation in training such as pinching/grasping and transporting an object (e.g., task-oriented training). Furthermore, repetitive finger movements are also challenging because of spasticity and abnormal muscle co-contraction.

In recent years, robot-assisted training has garnered attention as an effective intervention for increasing the training dose, even in individuals with stroke with severe motor impairments. Robot-assisted training allows for self-training without special assistance and/or guidance by clinicians after completing the initial setting, enabling repetitive and long-term training post-stroke. Another strength of robot-assisted training is that the difficulty level (for example, the amount of assistance) can be controlled to match the remaining motor functions. This adaptability enables individuals with severe motor impairments to engage in repetitive training. A previous study showed that robot-assisted training with usual rehabilitation improves motor function compared with usual rehabilitation alone.^11^ However, there are fewer rehabilitation robotic devices for the distal upper limb (wrist and hand) than for the proximal upper limb (shoulder and elbow) with evidence based on randomized controlled trials and a stable supply from distributors.^12,13^ In addition, many rehabilitation robotics are large and heavy, complicating their transport and limiting their use, particularly for individuals with stroke who require mobility assistance or are living at home.

To address this challenge, we developed a novel end-effector robot that provides intensive finger movement training. The robot is easy to carry and can therefore be used in hospital rooms and at home. This preliminary single-arm study aimed to investigate the effects of robot-assisted repetitive finger movement training on the hand motor function in individuals with post-stroke hemiparesis.

## Materials and Methods

### Study design and participants

This study was designed as a prospective single-arm intervention study. The study protocol was approved by the Ethics Review Committee of Fujita Health University (approval no. HM25-089) and registered it in the UMIN Clinical Trials Registry (UMIN no. UMIN000050491).

Eleven individuals with subacute stroke (mean age, 62.5 years; standard deviation, 16.2, three females) were enrolled in the rehabilitation ward in Fujita Health University Hospital between May 2023 and April 2024. All participants provided written informed consent before participation, in accordance with the Declaration of Helsinki of 1964, as revised in 2013. The inclusion criteria were as follows: 1) individuals who had experienced a stroke (either first or recurrent) and were aged ≥20 years; 2) a Stroke Impairment Assessment Set for Finger Function (SIAS-FF) score of 1A or more (minimal voluntary movement or mass flexion)^14^; 3) ability to sit independently with a backrest. The exclusion criteria were 1) inability to follow instructions or understand the training and 2) contractures in the wrist or hand that interfered with robot-assisted finger movement training.

### Finger rehabilitation robot

**Figure 1:**
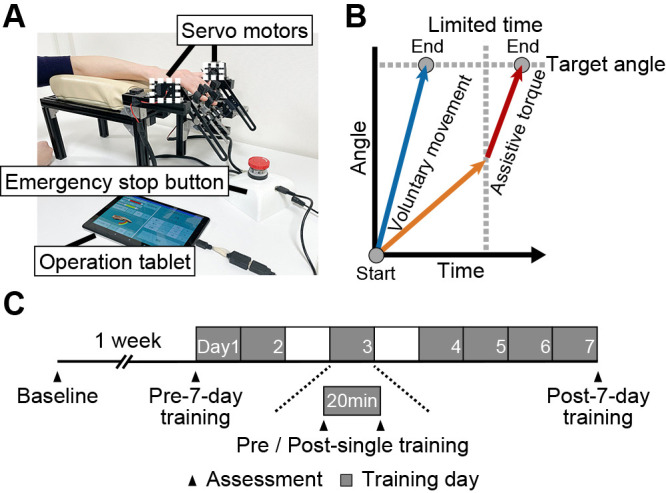
The novel end-effector robot and experimental protocol. (A) An overview of the robot. The repetitive finger movement is controlled by the rotation of the robot arm driven by two servo motors. The parameters of the robot operations (rotational speed of servo motors, target angle of movement, and number of repetitions) are adjusted using an operation tablet. There is an emergency stop button on the device. (B) Algorithm for providing assistive torque. In robot-assisted finger movement training, the participants move their fingers to reach the target angle within a limited time (blue arrow). Assistive torque (red arrow) is provided if a participant cannot move their fingers from the start to the target angle within a limited time (orange arrow). (C) An example of the experimental protocol. Each participant performed the robot-assisted training for 20 min (single training) on 7 non-consecutive days (7-day training). We assessed outcome measures before (at baseline and pre-7-day training) and after (post-7-day training) the 7-day training, as well as before and after the daily training (pre- and post-single training).

We developed a novel end-effector robot that provides intensive finger movement training (Figure 1A). The robot was designed to allow flexion and extension of the metacarpophalangeal (MP) and proximal interphalangeal (PIP) joints of the index to the little fingers. The repetitive finger movement was controlled by the rotation of the robot arm driven by two servo motors (angular velocity: 37.5–375.0 deg/s, torque: 0.049–0.490 Nm). The robot arm rotated downward during finger flexion and upward during finger extension. The participant’s fingers were attached to the device using rings made of plant-based resin, with magnets attached to the distal phalanges of the fingers. During robot-assisted finger movement training, the participants placed their forearms in a pronated position on a cushion, with their hands resting on a silicone pad. The height of the silicone pad was adjusted to align with the natural position of the wrist. To maintain the participant’s wrist in a neutral position, their forearm or wrist was held with a band. The connection point between the fingers and device and the position of the silicone pad were adjusted to match the length of each participant’s fingers and forearms. The device has two training modes: passive and active-assistive. In the passive mode, the robot moves the fingers within a set range of movements. In the active-assistive mode, the robot provides assistive torque if the voluntary finger movement does not reach the target angle within a limited time. Therefore, no assistive torque was provided if participants could move their fingers to reach the target angle within a limited time (Figure 1B). The assistive torque was provided linearly from the angle reached at the end of the limited time to the target angle, with an angular velocity of 375.0 deg/s and a torque of 0.490 Nm. There is an emergency stop button on the device that can be operated by the experimenter and the participant. It should be noted that this robot is not currently accessible through any public sources.

### Experimental protocol

The participants performed robot-assisted active-assistive finger flexion-extension movements for 20 min daily (single training) and repeated this training on 7 non-consecutive days (7-day training) (Figure 1C). All participants also received the usual rehabilitation training comprising 60 min of physical, occupational, and speech-language therapies (if needed) every day. Robot-assisted finger flexion-extension movements were repeated 10 times per set, and the sets were performed to maximum repetition for 20 min. The number of training days, repetitions per set, and movement duration per day were selected in an exploratory manner as a preliminary investigation. The participants were required to move their fingers to the target angle within a limited time. The target angle was set to the maximum passive movement range for each participant. The experimenter adjusted the number of repetitions and the limited time for the flexion and extension phases separately (time until assistive torque was provided) by considering each participant’s performance and fatigue. In particular, the limited time was set to be as short as possible. Although the limited time was not displayed to the participants, they were informed that the assistive torque would be provided if they were unable to reach the target angle.

### Outcome measures

All assessments were conducted by one of the experimenters, an occupational therapist with 11 years of clinical experience (KU).

### Active range of motion (AROM)

To investigate the effect of robot-assisted finger movement training on hand motor function, we assessed the active maximum flexion and extension angles of the MP and PIP joints at three timepoints: 1 week before the first day of the 7-day training (baseline), immediately before and after the 7-day training (inter-day effect), and before and after the training each day (intra-day effect).

During the assessment, the participants sat on a chair or wheelchair with their forearms in the neutral position and their hands placed on a table with the shoulder slightly flexed, abducted, and at approximately 90° of elbow flexion. The wrist was held in a custom-designed holder to maintain the forearm in the neutral position as possible. The participants were asked to perform three repetitive maximum flexion-extension finger movements twice at a comfortable speed with 1 min intervals between sets (i.e., three repetitive flexion-extension finger movements×two sets). The movement was recorded using a two-dimensional camera (frames per second: 60 or 240; resolution: 2340×1080) mounted on a tripod approximately 45 cm away from the hand in the sagittal plane. We placed color stickers (8 mm in diameter) at the distal interphalangeal (DIP) joint, PIP joint, MP joint, and the midpoint of the metacarpal. We analyzed only the index finger because the middle to little fingers were hidden during movement and could not be monitored when recording from the sagittal plane.

We performed pose estimation using DeepLabCut™ ^15^ to calculate the AROM. We extracted frames offline from the video data for analysis and manually labeled the parts where the markers were placed. Marker positions were identified in the video using deep learning based on the labeled data. We calculated the maximum flexion and extension angles for each of the six flexion-extension movements and the average between them offline using a custom program written in MATLAB (MathWorks, Inc., Natick, MA, USA).

### Electrophysiological assessment

Electromyography (EMG) was performed during the AROM assessment to evaluate the co-contraction index (CCI) of the flexor digitorum superficialis (FDS) and extensor digitorum muscles (EDM). The CCI has previously been used as a proxy for the amount of muscle co-activation during voluntary movement in individuals after stroke.^16^

Pairs of adhesive Ag/AgCl surface EMG electrodes (NM-31; Nihon Kohden Corp., Tokyo, Japan) were placed on the belly of each muscle, identified through manual palpation, and the skin was abrased with gel and cleaned with rubbing alcohol. The EMG activities were recorded at 2,000 Hz using a biosignal recording system (Nurropack X1 MEB-2312; Nihon Kohden Corp., Tokyo, Japan), with band-pass filtering from 10 to 500 Hz.^16–18^ The recorded data were digitized using a Micro 1401 AD converter (Cambridge Electronic Design, Ltd., Cambridge, England) and stored on a computer.

All analyses were performed offline using a custom program written in MATLAB. The raw EMG signals were first subtracted from the mean of the raw EMG to correct the offset of the baseline, and the root mean square (RMS) of the EMG in each muscle was calculated using a 500-ms window. The RMS of each muscle in each set during repetitive movements (three repetitive flexion-extension finger movements=one set) was normalized by the peak RMS of each set (EMG_EDM_ and EMG_FDS_) and used to calculate the CCI. The CCI was calculated separately for the flexion and extension phases. Flexion and extension phases were separated through visual inspection. The CCI (for the three flexion phases in the three flexion-extension movements) was calculated as follows^19^:


$$\text{CCI}\;[\%]=\frac{{2I}_{\text{EDM}}}{I_{\text{total}}}\times 100$$

where *I_EDM_* is the total EMG_EDM_ activity calculated using the following equation:


$$I_{\text{EDM}}=\int_{t_{1}}^{t_{2}}{\text{EMG}}_{\text{EDM}}\;\left(t\right)\text{dt}$$

where t_1_ to t_2_ indicates the period from initiation to termination of the flexion phase. *I_tatal_* is the total EMG_EDM_ and EMG_FDS_ activity, which was calculated using the following equation:


$$I_{\text{total}}=\int_{t_{1}}^{t_{2}}\left[{\text{EMG}}_{\text{EDM}}+{\text{EMG}}_{\text{FDS}}\right]\;\left(t\right)\text{dt}$$

We calculated the CCI for each set and used the average between the two sets as a representative value.

### Clinical assessment

We assessed the SIAS-FF and the modified Ashworth scale (MAS)^20^ at three timepoints, namely baseline, pre-7-day training, and post-7-day training, to investigate the effect on hand motor impairment and function. We assessed the MAS of the wrist and finger flexors (the MP and PIP joints).

### Statistical analyses

Two participants were excluded from the CCI analysis because one had missing data owing to a technical error, and the other showed continuous rather than phasic muscle activity, preventing the separation of the flexion and extension phases through visual inspection.

We performed a two-way repeated-measures analysis of variance for the CCI with within-participant factors of time (pre- and post-single training) and day (Days 1 to 7) to analyze the intra-day effect. To examine the inter-day total training effect, we performed a one-way repeated-measures analysis of variance for the CCI and Friedman’s test for SIAS-FF and MAS with within-participant factors of time (baseline, pre-7-day training, and post-7-day training). Notably, we did not perform a statistical analysis for the AROM because the accurate joint angle could not be estimated for some participants’ data (24.0% of the dataset among all participants) owing to certain measurement issues: the covering of the DIP and/or PIP joints by the thumb at maximum flexion or parallax error, which occurred because the forearm pronated or supinated during the measurement, limiting information acquisition.

All statistical analyses were performed using SPSS version 26 (IBM Corp., Armonk, NY, USA). The effects were considered statistically significant at *p*<0.05.

## Results

### Participants

The participants’ characteristics are presented in Table 1. Ten participants completed the 7-day training. One participant dropped out after the second training day because they wished to focus only on the usual rehabilitation. No adverse events occurred during the 7-day training. The mean number of daily repetitions of finger flexion-extension movements during the single training was 82.0 (range, 30–120).

### AROM

**Figure 2:**
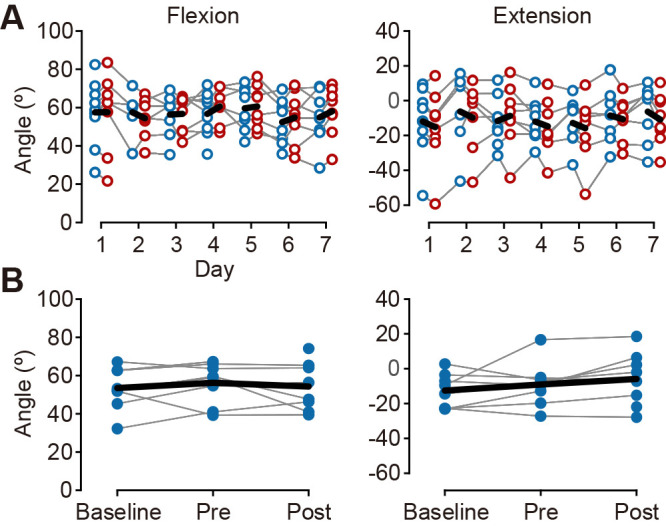
Changes in the active range of motion of the metacarpophalangeal joint of the index finger. (A) The intra-day effect. Blue and red circles represent individual data at the pre- and post-single training. Black thick lines indicate the mean values of all participants. (B) The inter-day effect. Each plot with blue dots shows individual data at baseline, pre-7-day training, and post-7-day training (“Pre” and “Post”). Black lines indicate the mean values of all participants.

We calculated the AROM for the MP joint only because the estimation of the AROM of the PIP joint was impossible in many participants for whom the DIP and PIP joints were hidden by the thumb during maximum flexion. Qualitative observations revealed that the maximum flexion angle tended to increase after the single training on the fourth training day, whereas the maximum extension angle tended to decrease after each single training throughout the training period (Figure 2A). There were no evident changes in the inter-day effect after the 7-day training for the maximum flexion or extension angles (Figure 2B).

### Electrophysiological assessment

**Figure 3:**
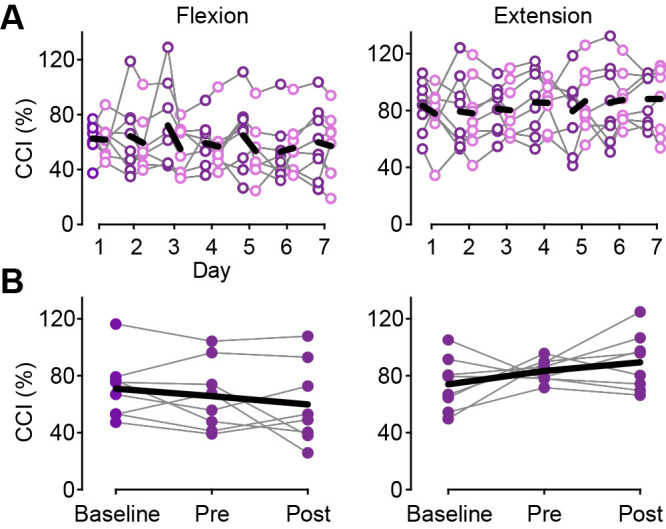
Changes in the co-contraction index. (A) The intra-day effect. Purple and pink circles represent individual data at the pre- and post-single training. Black thick lines indicate the mean values of all participants. (B) The inter-day effect. Each plot with purple dots shows individual data at baseline, pre-7-day training, and post-7-day training (“Pre” and “Post”). Black lines indicate the mean values of all participants.

For the intra-day effect, the CCI was significantly decreased after each single training only in the flexion phase (Figure 3A, Table 2). To better understand whether the decreased CCI was attributable to decreased muscle activity in the antagonist muscle (i.e., EMG_EDM_) or increased activity in the agonist muscle (i.e., EMG_FDS_), we additionally examined the activity levels in each muscle. The EMG_EDM_ was significantly decreased after each single training, whereas the EMG_FDS_ showed marginal changes (Supplementary materials). This indicates that the decrease in antagonist muscle activity may contribute to the decreased CCI that was observed in the flexion phase. For the inter-day effect, the CCI did not change during the three timepoints in the flexion or extension phases (Figure 3B, Table 2). These results indicate that robot-assisted finger movement training immediately improved the CCI after each single training; that is, there was a decrease in abnormal muscle co-activation. However, there was no carry-over effect across days, resulting in no significant change after the 7-day training.

### Clinical assessment

The SIAS-FF did not significantly change after the 7-day training (χ^2^ [2]=4.67, *p*=0.10) (Table 3). The MAS of the wrist did not change at any timepoint. As the variance of the data was zero, we did not perform a statistical analysis. The MAS of the finger did not change after the 7-day training (χ^2^ [2]=3.00, *p*=0.22).

## Discussion

This preliminary single-arm study investigated the intra- and inter-day effects of novel robot-assisted finger movement training on hand motor function and muscle activation patterns in individuals with post-stroke hemiparesis. The AROM tended to increase and the muscle co-activation between the agonist and antagonist muscles during voluntary finger flexion significantly decreased after each single training; however, there were no significant changes after the 7-day training. The training had no significant inter-day effects on the SIAS-FF and MAS.

Stroke usually damages motor pathways in the brain, causing motor paresis and abnormal co-activations of the upper limb muscles across segments and between the agonist and antagonist muscles within a segment.^21^ These abnormal co-activations limit active range of movement and functional control.^22,23^ Furthermore, some individuals with stroke who experience finger paresis tend to perform finger flexion and extension with compensatory tenodesis action with wrist extension and flexion, resulting in augmented co-activation patterns between the agonist and antagonist muscles. Therefore, improving finger paresis and abnormal co-activation is thought to be the key to enhancing hand motor function after stroke. The present study showed that robot-assisted finger movement training significantly improved the co-activation between the agonist and antagonist muscles immediately after a single training, specifically during finger flexion movement; there was a decrease in the muscle activity in the finger extensor (EMG_EDM_) with no change in the activity level of the finger flexor (EMG_FDS_). Because no previous studies have provided reference values for the CCI between the finger flexors and extensors in healthy individuals or individuals with stroke, the present findings cannot be compared with previous studies. However, these results may be explained by physiological mechanisms such as the reciprocal inhibition of the agonist to antagonist muscles. Reciprocal inhibition is a system in which, when the agonist muscle contracts, Ⅰa afferents originating from the muscle spindle primary endings of the agonist muscle activate Ⅰa inhibitory interneurons projecting to the motor neurons of the antagonist muscle, inhibiting the muscle activity of the antagonist muscles and allowing efficient voluntary movement.^24^ A previous study has reported that the function of reciprocal inhibition is decreased owing to a decrease in the excitability of interneurons, which is thought to be caused by a reduction in descending inputs to the interneurons.^25^ The present finding of decreases in the antagonist muscle during voluntary finger flexion suggests that repetitive finger flexion-extension movements mediated by the robot may immediately improve the reciprocal inhibitory mechanism, likely through an increase in the excitability of the Ⅰa inhibitory interneurons *per se* or that of the muscle spindle.

Qualitative observations revealed that the maximum flexion angle tended to increase after each single training from the middle of the training period (i.e., after the fourth training day), while the maximum extension angle did not increase during the training period (Figure 2A). Notably, similar to the co-activation patterns, improvements in the AROM were observed only during flexion and not during the extension phases. There are several possible explanations for these phenomena. One possible explanation is related to the differences in the amount of assistance provided during finger movements. In the present robot-assisted finger movement training, finger flexion was performed in the direction of gravity, whereas extension was directed against gravity. This implies that only finger flexion movements had additional assistance related to gravity. Previous studies have reported that the amount of assistance provided is crucial to increasing the effectiveness of robot-assisted training for individuals with stroke, and that substantial assistance is particularly essential for those with severe motor impairments.^26,27^ Given that many participants in the present study had severe motor impairments, the improvements in the AROM and co-activation patterns during the flexion phase may be attributed to the relatively greater gravity-related assistance, which enabled adequate repetitive voluntary finger flexion. Another possible explanation is the difference in recovery patterns between flexion and extension movements after a stroke. Motor recovery after stroke is gradual and usually starts with the restoration of flexion movement, followed by extension movement.^28^ This pattern suggests that performing finger flexion is less challenging than executing finger extension for individuals with stroke with moderate-to-severe motor impairment.^29^ These findings may explain our findings that improvements in the AROM and co-activation patterns were observed only during flexion.

Regardless of the intra-day effects of the training, there were no significant improvements in the outcome measures (i.e., AROM, muscle co-activation patterns, SIAS-FF, or MAS) after completing the 7-day training. This indicates that the present robot-assisted finger movement training does not have a significant carry-over effect. There are two possible explanations for these findings. First, the training dose in this study (i.e., 30–120 repetitions/day, 20 min/day for 7 days, equivalent to a total of 140 min) may have been insufficient to improve hand motor function. The number of repetitions per day varied among participants, with those performing 50 or fewer repetitions per day often affected by minor device malfunctions and health-related issues (Supplemental materials). A previous study has reported that high-intensity robot-assisted training (i.e., 600–800 repetitions/day, 5 days/week for 4 weeks) improves upper limb motor function compared with the effect of low-intensity intervention (150–200 repetitions/day).^30^ Other studies have reported that upper limb motor function improves with robot-assisted training performed for 480–900 min.^31,32^ Therefore, the training dose in the present study may be considered insufficient, resulting in no evident improvements in motor function after the 7-day training. However, it should also be noted that the severity of the impairment was not comparable. Many of the present study participants had severe motor impairments; however, those in previous studies had moderate impairments. Individuals with stroke with severe motor impairments are known to have poor improvements in motor function.^33^ Thus, in such cases, the intervention effects may be insufficient to produce significant improvements within a given intervention period. Second, the lack of an evident carry-over effect after the 7-day training may be related to the characteristics of the clinical assessment items used in the present study. For example, the SIAS-FF is used to assess the level of individuation of fingers, which is not comparable to the movement in the present robot-assisted training, which comprised repeated mass flexion and extension of the index to the little fingers. However, the intra-day effects on the muscle co-activation patterns observed in the present study suggest that the present robot-assisted training may be useful as a preconditioning intervention before performing complex functional training because robot-assisted training immediately led to the selective activation of the agonist muscles during finger movements. This control state allows for more functional and/or complex task training of the upper limb, which requires dexterous finger motor functions, such as pinching/grasping and transporting an object.

The present study had some limitations. First, we implemented a single-arm design as a preliminary investigation with no control conditions or groups, making it difficult to distinguish the effects of usual rehabilitation from those of robot-assisted finger movement training. A future study with a high training dose (high number of repetitions and long duration of training) and a control condition/group is required to verify the effects of robot-assisted training. Second, there was bias in the participants’ hand motor functions. Many participants who completed the 7-day training had severe motor impairments. Therefore, these results cannot be generalized to individuals with stroke of various motor function severities. Given that individuals with moderate motor impairments demonstrate greater improvement after robot-assisted training than those with severe impairments,^30^ a change in the severity of the target patients may result in different outcomes after the present robot-assisted training. Finally, several outcome measures could not be analyzed. Quantitative analysis was not performed for the AROM because of inaccurate joint angles in some participants owing to certain measurement issues (Statistical analyses section). Future studies should address this limitation by conducting three-dimensional analysis using multiple cameras. Furthermore, although the present study demonstrated positive intra-day effects, the potential mechanism underlying this improvement remains unclear. Future studies are required to clarify the potential mechanism underlying the improvement in muscle co-activation patterns.

## Conclusions

The present novel robot-assisted repetitive finger movement training improved muscle co-activation patterns as an intra-day effect, demonstrating its potential as a preconditioning intervention to better support functional finger movement control. Future studies with high training doses are required to further examine the effects of this robot-assisted training.

## Figures and Tables

**Table 1:** Participants’ characteristics

Variable	N=11
Age, mean (SD)	63.4 (15.7)
Sex, male/female, *n*	8/3
Days after stroke onset, mean (SD)	47.5 (17.5)
Type of stroke, ischemic/hemorrhagic, *n*	8/3
Affected side, left/right, *n*	5/6

SD, standard deviation

**Table 2:** Intra- and inter-day effects on the co-contraction index

Factor	Flexion				Extension		
	df	*F*	*p*		df	*F*	*p*
Intra-day effect							
Time	1, 7	7.30	0.03		1, 7	0.11	0.75
Day	6, 42	0.56	0.76		6, 42	0.90	0.51
Time×Day	6, 42	1.66	0.15		6, 42	0.72	0.64
Inter-day effect							
Time	2, 14	1.31	0.30		2, 14	1.62	0.23

**Table 3:** Changes in the Stroke Impairment Assessment Set for Finger Function and modified Ashworth scale

ID	SIAS-FF				MAS						
					Wrist flexors				Finger flexors (MP and PIP joints)		
	Baseline	Pre	Post		Baseline	Pre	Post		Baseline	Pre	Post
1	1c	1c	1c		1+	1+	1+		1+	1+	1+
2	1c	1c	1c		1+	1+	1+		1	1	1
3	1c	1c	1c		1	1	1		1	1	0
4	1b	1c	1c		1	1	1		0	0	0
5	1c	1c	1c		1	1	1		0	0	0
6	2	2	3		0	0	0		0	0	0
7	1c	1c	1c		1	1	1		1	1	1
8	1c	1c	1c		1	1	1		0	1	0
10	1c	1c	2		1	1	1		0	0	0
11	1c	1c	1c		1	1	1		1	1	1

SIAS-FF, Stroke Impairment Assessment Set for Finger Function; MAS, modified Ashworth scale; Pre, Pre-7-day training; Post, Post-7-day training; MP, metacarpophalangeal; PIP, proximal interphalangeal. Note that ID9 was excluded from the analysis as this participant withdrew from the study.
