# Supplementary material for: Intra- and inter-day effects of novel robot-assisted hand movement training in individuals with post-stroke hemiparesis: a single-arm pilot study
Source: Fujita Med J. 2026 Feb 28;12(2):114–20. doi: 10.20407/fmj.2025-025 (PMC13129707; doi:10.20407/fmj.2025-025)
Supplement: Supplementary file 1 — Supplementary materials [file fmj-12-114_s1.pdf]

Supplemental material

Given that the co-contraction index (CCI) significantly decreased after each single training, we performed an additional analysis to confirm whether the decrease was attributable to decreased muscle activity in the antagonist muscle (i.e., EMG_{EDM}) or increased activity in the agonist muscle (i.e., EMG_{FDS}). We performed a two-way repeated-measures analysis of variance for each muscle activity with within-participant factors of time (pre- and post-single training) and day (Days 1 to 7).

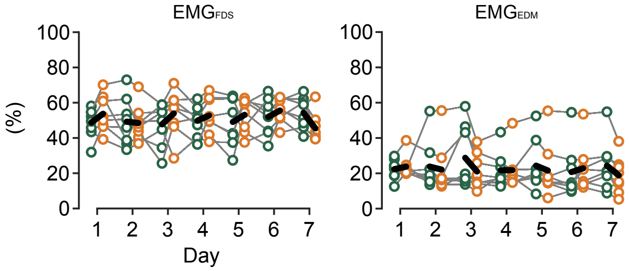

Supplementary Figure 1. Change in EMG_{FDS} and EMG_{EDM} in the flexion phase.

Green and orange circles represent individual data at the pre- and post-single training. Black thick lines indicate the mean values of all participants.

Supplementary Table 1. EMG_{FDS} and EMG_{EDM} in the flexion phase.

	EMG_{FDS}			EMG_{EDM}		
**Factor**	**df**	***F***	***p***	**df**	***F***	***p***
Time	1, 7	1.13	0.32	1, 7	12.06	0.01
Day	6, 42	0.80	0.58	6, 42	0.29	0.94
Time × Day	6, 42	1.91	0.10	6, 42	3.34	0.01

FDS, flexor digitorum superficialis; EDM, extensor digitorum muscles.

Supplementary Table 2. Number of daily repetitions of robot-assisted training.

	Training days						
**ID**	**1**	**2**	**3**	**4**	**5**	**6**	**7**
1	39	66	86	80	80	80	80
2	70	90	90	89	84	78	77
3	63	90	30	70	80	50	50
4	80	90	100	100	92	100	100
5	80	80	70	80	90	90	90
6	100	103	100	100	105	110	113
7	67	69	76	80	90	93	90
8	70	60	73	80	80	60	70
10	61	90	100	50	100	110	120
11	90	66	90	90	90	100	100

Note that ID9 was excluded from the analysis as this participant withdrew from the study.
